# Effects of 6-months' Exercise on Cardiac Function, Structure and Metabolism in Female Hypertensive Rats–The Decisive Role of Lysyl Oxidase and Collagen III

**DOI:** 10.3389/fphys.2017.00556

**Published:** 2017-08-03

**Authors:** Rolf Schreckenberg, Anja-Maria Horn, Rui M. da Costa Rebelo, Sakine Simsekyilmaz, Bernd Niemann, Ling Li, Susanne Rohrbach, Klaus-Dieter Schlüter

**Affiliations:** ^1^Physiologisches Institut, Justus-Liebig-Universität Giessen Giessen, Germany; ^2^Institut für Pharmakologie und Klinische Pharmakologie, Universitätsklinikum Düsseldorf Düsseldorf, Germany; ^3^Klinik für Herz-, Kinderherz- und Gefäßchirurgie, Universitätsklinikum Giessen Giessen, Germany

**Keywords:** arterial hypertension, aerobic exercise, cardiac function, lysyl oxidase, osteopontin, cardiac fibrosis, cardiac hypertrophy

## Abstract

**Purpose:** According to the current therapeutic guidelines of the WHO physical activity and exercise are recommended as first-line therapy of arterial hypertension. Previous results lead to the conclusion, however, that hearts of spontaneously hypertensive rats (SHR) with established hypertension cannot compensate for the haemodynamic stresses caused by long-term exercise. The current study was initiated to investigate the effects of aerobic exercise on the cardiac remodeling as the sole therapeutic measure before and during hypertension became established.

**Methods:** Beginning at their 6th week of life, six SHR were provided with a running wheel over a period of 6 months. Normotensive Wistar rats served as non-hypertensive controls.

**Results:** In Wistar rats and SHR, voluntary exercise led to cardioprotective adaptation reactions that were reflected in increased mitochondrial respiration, reduced heart rate and improved systolic function. Exercise also had antioxidant effects and reduced the expression of maladaptive genes (TGF-β1, CTGF, and FGF2). However, at the end of the 6-months' training, the echocardiograms revealed that SHR runners developed a restrictive cardiomyopathy. The induction of lysyl oxidase (LOX), which led to an increased network of matrix proteins and a massive elevation in collagen III expression, was identified as the underlying cause.

**Conclusions:** Running-induced adaptive mechanisms effectively counteract the classic remodeling of hearts subject to chronic pressure loads. However, with sustained running stress, signaling pathways are activated that have a negative effect on left ventricular relaxation. Our data suggest that the induction of LOX may play a causative role in the diagnosed filling disorder in trained SHR.

## Introduction

Arterial hypertension is of central importance as a direct risk factor for the development of numerous cardiovascular diseases. A number of primary and secondary risk factors are also indirectly responsible for a deterioration in the cardiovascular risk profile due to an increase in blood pressure (BP). Accordingly, there is a clear need for systematic antihypertensive therapy for the prevention of cardiovascular events and sequelae.

The current therapy guidelines from the WHO and the German Hypertension League recommend physical activity and sport as the first-line therapy—without, however, specifying individual therapy plans (Chobanian et al., 2003; WHO, 2003; Deutsche Hochdruckligae, 2008). Unlike drug intervention, there is little information available regarding the effects of physical activity and sport on the cardiovascular system in cases of existing hypertension (Schlüter et al., 2010).

The results of a number of studies based on animal experiments suggest that senescent rats with established hypertension do not compensate for additional running-induced hemodynamic stresses and therefore cannot benefit from the associated cellular adaptation mechanisms (Schultz et al., 2007; da Costa Rebelo et al., 2012). Prominent among the clinical and pathomorphological results of these studies is significant cardiac fibrosis, which primarily affects relaxation and thus diastolic function. Massive performance-induced myocardial hypertrophy and reduced calcium handling also lead to a detrimental functional and structural remodeling of the hearts. The stimulation of the sympathetic nervous system with subsequent activation of the renin-angiotensin system (RAAS) as well as induction of pro-fibrotic mediators such as transforming growth factor-β (TGF-β) and biglycan are largely responsible for these effects (O'Keefe et al., 2012; van de Schoor et al., 2016).

On the other hand, there are results that can be viewed as desirable adaptation reactions to aerobic endurance training: past studies have described the induction of antiapoptotic genes (Lajoie et al., 2004), improved mitochondrial respiration (Chicco et al., 2008), a reduction in free radicals (Bertagnolli et al., 2006), as well as a significant reduction in resting heart rate (HR) (Lee et al., 2006).

The precise circumstances and causes that have led to these heterogeneous results in the various hypertensive animal models are the subject of our research. Consequently, it is not yet possible to make concrete statements about the factors or mechanisms that determine the therapeutic success of endurance training and sport in patients with hypertension (Thompson et al., 2007).

To what extent physical training alone contributes to a fall in BP or prevents the development of arterial hypertension is still largely unknown (Pagan et al., 2015; Sharman et al., 2015).

In a prospective study, Allesøe et al. determined the risk of 12,093 female volunteers developing ischaemic heart disease (IHD) under the combined influence of physical activity and arterial hypertension. Hypertensive patients who completed a high level of daily physical activity had a three-fold higher risk of developing IHD but the underlying pathological mechanisms are unclear (Allesøe et al., 2016).

Our current project is primarily aimed at investigating the effects of a 6-months' running wheel training program on the cardiac remodeling of young, 1½-month-old spontaneously hypertensive rats (SHR) before and during hypertension became established.

Accordingly, the following hypothesis was formulated: Starting the physical training at a time when no hypertension-induced structural and metabolic dysfunctions are yet observed in the cardiovascular system should counteract the development of hypertension as a result of intact adaptation mechanisms and should thus have a long-term positive effect on the functional and structural remodeling of the hearts.

In addition to hypertrophy, the characteristic findings for the left ventricular myocardium that is subject to chronic pressure loads in SHR include excess production of radicals and remodeling of the extracellular matrix with the involvement of TGF-β, lysyl oxidase (LOX) and structure-forming proteins (Rysä et al., 2005; Brooks et al., 2010).

Training data such as the distance and time run and the speed were recorded continuously throughout the experiment; any changes in the systolic and diastolic BP and the HR were also recorded.

We have been able to show for the first time that endurance training has a positive effect on the classic remodeling of a heart that has been subject to a chronic pressure load but over the long term this adaptive response causes a massive induction of LOX through alternative signaling pathways, which results in a clinically relevant filling disorder of the left ventricular myocardium.

## Materials and methods

The investigation conforms the Guide for the Care and Use of Laboratory Animals published by the US National Institute of Health (NIH Publication No. 85–23, revised 1996). The study was approved by the local authorities for animal experiments (V54–19c 2015h 01 GI 20/1 No. 77/2014).

### Animals and exercise model

At the start of the 6th week of life, 6 female SHR were provided with a running wheel over a period of 6-months'. Normotensive female Wistar rats were used as non-hypertensive controls and were also allocated to 6 cages with running wheels. Both groups were supplemented by corresponding “non-running” control groups that were kept under identical conditions without access to running wheels.

The health status of the experimental animals was determined weekly using a “distress score” (Lloyd and Wolfensohn, 1999). Over the entire experimental period no animals had to be eliminated from the experiment based on the exclusion criteria of the score.

### Determination of the BP and HR

The systolic and diastolic BP and the HR were initially measured weekly and then subsequently every 2 or 4 weeks using non-invasive tail-cuff BP measurement. Prior to the start of the experiment the animals were adjusted to the experimental procedure over a week. The median of 10 consecutive measurements was calculated for each parameter described.

### Preparation of the heart

At the end of the experimental period rats were anesthetized by isoflurane inhalation. After cervical dislocation hearts were isolated and perfused in Langendorff technique to remove blood contamination.

### RNA isolation and real time RT-PCR

Total RNA was isolated from left ventricular tissue using peqGold TriFast (peqlab, Biotechnologie GmbH, Germany) according to the manufacturer's protocol. To remove genomic DNA contamination, isolated RNA samples were treated with 1 U DNase/μg RNA (Invitrogen, Karlsruhe, Germany) for 15 min at 37°C. One μg of total RNA was used in a 10 μl reaction to synthesize cDNA using Superscript RNaseH Reverse Transcriptase (200 U/μg RNA, Invitrogen, Karlsruhe, Germany) and oligo dTs as primers. RT reactions were performed for 50 min at 37°C. Real-time quantitative PCR was performed using MyiQ® detection system (Bio-Rad, Munich, Germany) in combination with the iTaq Universal SYBR Green Real-Time PCR Supermix (Bio-Rad, Munich, Germany). Quantification was performed as described before (Livak and Schmittgen, 2001). Primer sequences are listed in Supplementary Table 1.

### Picrosirius red staining

Samples were embedded with Tissue-Tek® (Sakura, Alphen, Netherlands) and sectioned in 10 μm slices. Histological sections were fixed in Bouin solution and subsequently stained in 0.1% (wt/vol) Sirius red solution (Sigma-Aldrich Chemie, Steinheim, Germany). Sections were washed by 0.01 N HCl, Aqua dest. and counterstained for nuclei by Mayers hemalaun solution, washed with Aqua dest. for 5 min and dehydrated with ethanol. Finally, histological slices were visualized under light microscopy. Total collagen content was quantified by digital image analysis using Leica Confocal Software Lite Version (LCS Lite). The mean of *n* = 6 preparations was used to quantify the extent of interstitial fibrosis.

### Microrespirometry

Immediately before oxygraphic measurements muscle fibers were permeabilized 30 min with saponin. After permeabilization the fibers were washed to remove saponin and adenine nucleotides. We used the high-resolution OROBOROS® oxygraph, a two chamber respirometer with a Peltier thermostat and integrated electromagnetic stirrers. The measurements were performed at 30°C in 1.42 ml incubation medium using different substrates: 10 mM pyruvate + 2 mM malate and 10 mM succinate + 5 μM rotenone. The weight specific oxygen consumption was calculated as the time derivative of the oxygen concentration (DATGRAPH Analysis software, OROBOROS®). The rate of state 3 respiration was determined following the addition of 5 mM ADP. State 4 respiration was measured after the addition of 1.8 mM atractyloside. The respiratory control index was calculated as ratio between state 3 to state 4 respiratory rates for each addition of ADP. For the analysis of uncoupled respiration, 2,4-dinitrophenol was added in a two-step titration up to 60 μM. The difference between atractylate-and antimycin A respiration (45 μM antimycin A) indicates the leak respiration (Niemann et al., 2010).

### Measurement of superoxide ($O_{2}^{-•}$)

To perform DHE staining, cryosections of the left ventricle (LV) were incubated with DHE (dissolved in 1 X PBS) for 10 min at 37°C in a light-protected humidity chamber, then fixed with Dako Fluorescent Mounting Medium (Dako, North America Inc., USA). Slides were imaged by fluorescence microscopy (LSM 510 META; Carl Zeiss, Jena, Germany) using an excitation wavelength of 488 nm; emission was recorded at 540 nm (Nazarewicz et al., 2013). Analysis was performed by digital image analysis using Leica Confocal Software Lite Version (LCS Lite). The mean fluorescence intensity of *n* = 4 ventricles was used to quantify the extent of $O_{2}^{-•}$.

### Western blot

Total protein was extracted from LV using RIPA Buffer (Cell Signaling, Danvers, MA, USA) according to the manufacturer's protocol. Briefly, the homogenate was centrifuged at 14,000 g at 4°C for 10 min and supernatant was treated with Laemmli buffer (Sigma-Aldrich, Taufkirchen, Germany). Protein samples were loaded on NuPAGE Bis-Tris Precast gels (10%; Life Technology, Darmstadt, Germany) and subsequently transferred onto nitrocellulose membranes. Blots were then incubated with a rabbit polyclonal LOX antibody purchased from Merck Millipore (Darmstadt, Germany; product ABT112) or rabbit polyclonal Col-III antibody purchased from Novus Biologicals (Littleton, USA; product NB600-594). Secondary antibody (HRP-conjugated) directed against rabbit IgG was purchased from Affinity Biologicals (Ancaster, ON; Canada).

### Rat cardiac fibroblasts

For isolation of adult cardiac fibroblasts, the non-myocytes fraction obtained from the isolation of cardiomyocytes by the Langendorff method was utilized. Cardiac fibroblasts are enriched by a period of brief attachment (2 h) and subsequent removal of less adherent cells such as endothelial cells. Cardiac fibroblasts were maintained in DMEM supplemented with 10% FCS and 1% penicillin and streptomycin under an atmosphere of 5% CO_2_ in air at 37°C. After reaching 60–70% confluency, cardiac fibroblasts were trypsinized and transferred to 6-well plates (1 × 10^5^ cells / well). 24 h later LOX siRNA, OPN siRNA or control siRNA (FlexiTube siRNA, Qiagen) oligonucleotides were transfected to the cells at a concentration of 0.5 nmol/l with Lipofectamine® RNAiMAX (Invitrogen) according to the protocol of the manufacturer. 48 h after siRNA transfection, medium was replaced by serum-free medium and 2 h later cell treatment was initiated as indicated. These primary cultures are >95% fibroblasts at the time of confluency as judged by positive staining for vimentin and lack of staining for von Willebrand factor.

### Echocardiography

Rats were anesthetized by isoflurane inhalation (2% isoflurane, 98% O_2_) and left ventricular function was assessed by two-dimensional echocardiography using a 12.5-MHz probe (Vivid i, GE Health Care). All measurements were performed in accordance with the conventions of the American Society of Echocardiography and were conducted by the same trained, blinded sonographer. Left ventricular function was visually scanned by B-mode imaging in short and long parasternal axis. Measurement of left systolic and diastolic ventricular wall thicknesses and diameters as well as measurement of aortal and left atrial diameters was performed in long parasternal axis by M-Mode imaging. Fractional shortening (FS) was calculated as FS = (LVEDD-LVESD/LVEDD) × 100, where LVEDD and LVESD are LV internal diameters in end-diastole and end-systole, respectively. Fractional shortening (% FS) and left ventricular ejection fraction (% EF) were calculated from mean-values of 6 independently performed measurements per setting. Pulsed wave (PW) doppler was used to assess transmitral valve flow velocities during early (E) and atrial (A) filling periods.

### Statistics

Data are expressed as indicated in the legends. ANOVA and the Student-Newman-Keuls test for *post hoc* analysis were used to analyze experiments in which more than one group was compared. In cases in which two groups were compared, Student's *t*-test or Mann-Whitney-test was employed, depending of a normal distribution of samples (Levene-test). *P* < 0.05 was regarded as significant.

## Results

### Running performance and training-induced adaptations in cardiac and skeletal muscles

Over the entire training period the mean weekly running performance was 30.2 km for the Wistar and 51.9 km for the SHR. However, with an average speed of 3.2 km/h the Wistar significantly surpassed the speed of the SHR (2.5 km/h). The profile of the running performance is shown in Supplemental Figure 1.

**Figure 1:**
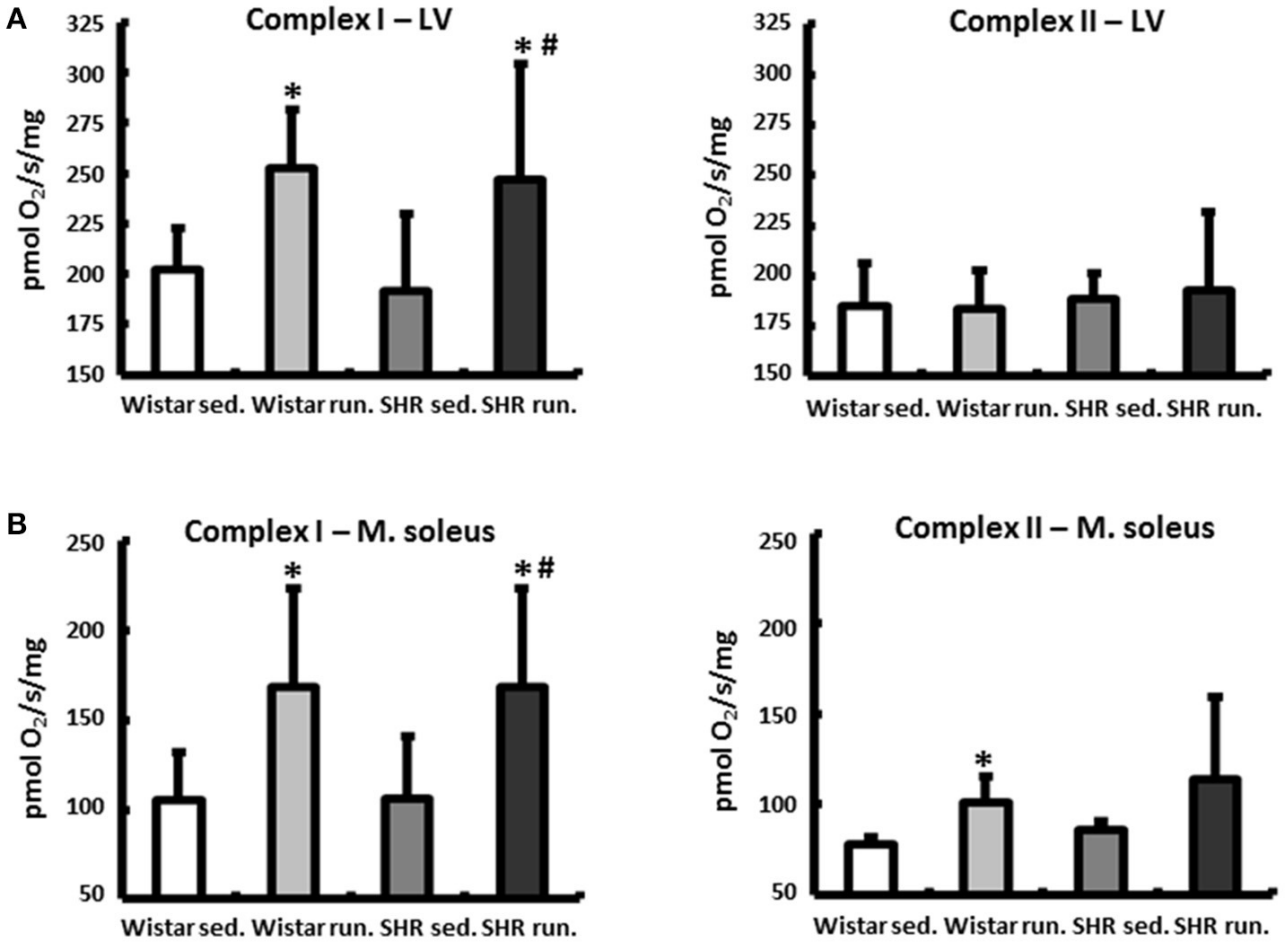
Microrespiratory measurements on permeabilized heart and skeletal muscle fibers. **(A)** Running training improved complex I respiration and did not influence complex II activity in the LV. **(B)** The efficiency of complex I as well as complex II respiration was increased in the soleus muscle of both running groups. Data are means ± S.D. of *n* = 6 animals. ^*^p < 0.05 vs. Wistar sed., ^#^*p* < 0.05 vs. SHR sed.

At the end of the experimental period the functional status of the mitochondria was analyzed using microrespiratory measurements on permeabilized heart and skeletal muscle fibers. The efficiency of the pyruvate respiration increased in the running Wistar by 25 ± 4% (LV) or 61 ± 10% (soleus muscle) compared to the non-running controls. In the trained SHR a comparable increase of 28 ± 9% (LV) and 59 ± 13% (soleus muscle) was observed. Succinate respiration, on the other hand, did not change in the cardiac muscle in either the Wistar or SHR. In the soleus muscle there was a training-induced increase of 30 ± 3% identified in Wistar and of +32 ± 11% in SHR (Figure 1).

**Figure 2:**
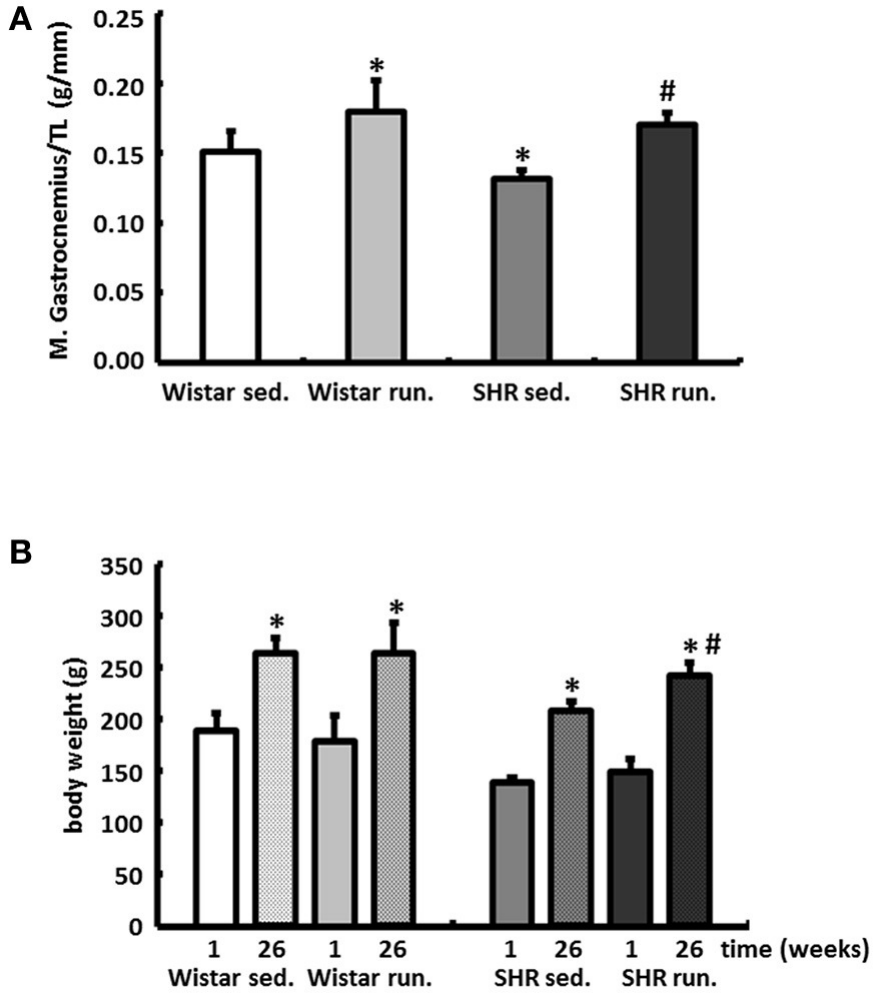
Effects of running training on muscle growth. **(A)** A comparable increase in muscle weight (red and white fraction of the gastrocnemius muscle) was found in both running groups. Data are means ± S.D. of *n* = 6 animals. ^*^p < 0.05 vs. Wistar sed., ^#^*p* < 0.05 vs. SHR sed. **(B)** Gains in BW could be observed in all groups over the study period, however, the largest increase could be determined in running SHR. Data are means ± S.D. of *n* = 6 animals. ^*^p < 0.05 vs. first week, ^#^*p* < 0.05 vs. 26th week SHR sed.

The weight of the skeletal musculature was also determined, indicating comparable individual training efficiency of Wistar and SHR. The weights of the gastrocnemius muscles (red and white fraction, normalized to the tibia length (TL)), representative of the muscle groups in the extremities, are shown in Figure 2A. The mean body weights of all treatment groups at the beginning and the end of the experimental period are shown in Figure 2B.

### Effects of running training on the development of BP and HR

**Figure 3:**
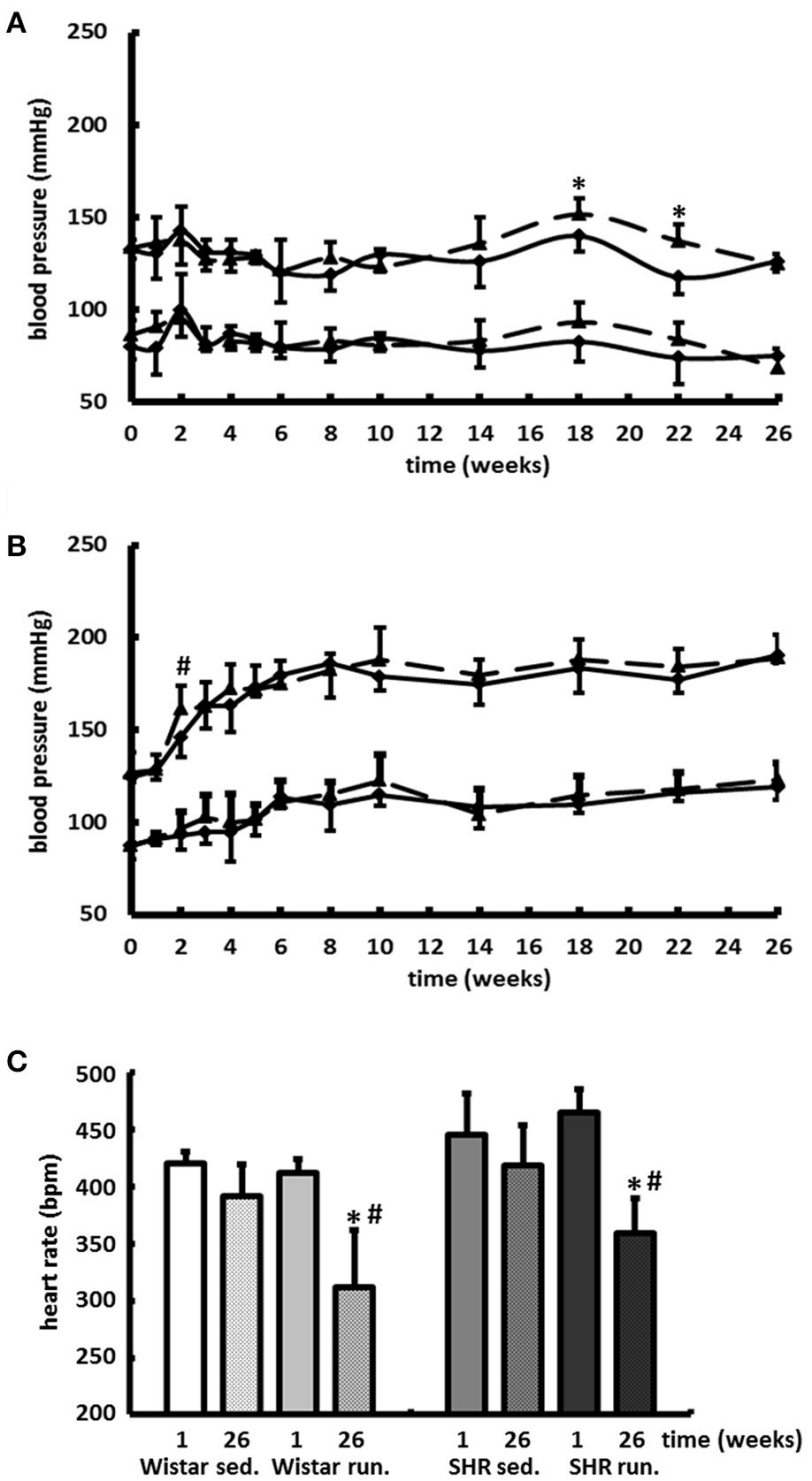
Development of the BP and the resting HR. Neither the temporal development nor the level of the systolic or diastolic BP were affected by 6-months' voluntary aerobic training in **(A)** Wistar rats and **(B)** pre-hypertensive SHR. Blood pressure values of non-runners are represented by a solid line, values of runners are represented by a broken line. Data are means ± S.D. of *n* = 6 animals. ^*^p < 0.05 vs. Wistar sed., ^#^*p* < 0.05 vs. SHR sed. **(C)** HR was lowered over the entire course of the training in the running animals compared to the non-running controls. Data are means ± S.D. of *n* = 6 animals. ^*^p < 0.05 vs. first week, ^#^*p* < 0.05 vs. 26th week sed.

Compared to the results for the corresponding non-running control groups, the running training did not affect either the temporal development or the level of the systolic or diastolic BP (Figures 3A,B). Only the resting pulse was lowered over the course of the training in the running animals compared to the non-running controls (Wistar −100 bpm, SHR −106 bpm, *p* < 0.05); see Figure 3C.

### Effects of training on the cardiac remodeling

**Figure 4:**
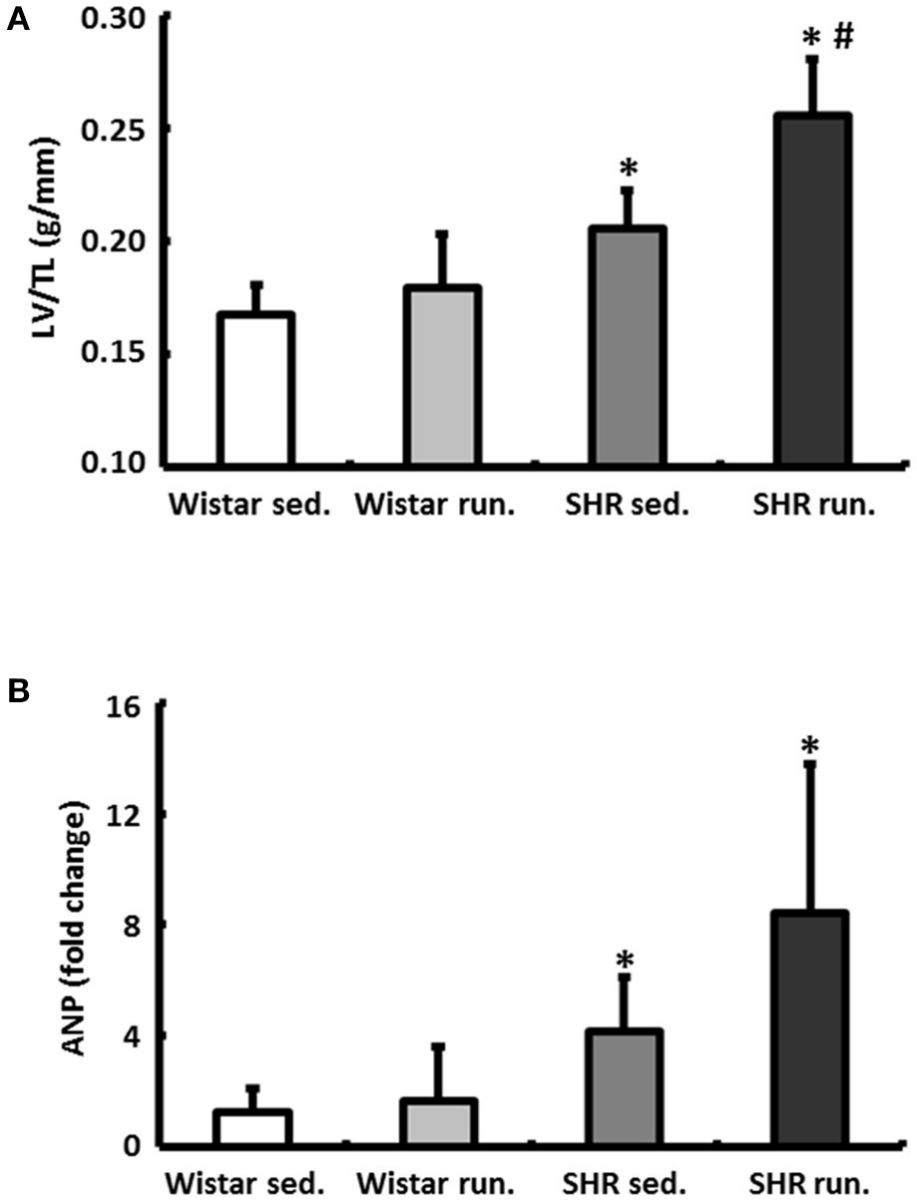
Effects of running training on cardiac hypertrophy. **(A)** The wet weight of the LV was normalized to the TL. **(B)** Left ventricular hypertrophy is also marked by the corresponding expression of ANP. Data are means ± S.D. of *n* = 6 animals. ^*^p < 0.05 vs. Wistar sed., ^#^*p* < 0.05 vs. SHR sed.

The wet weight of the LV was normalized to the TL of the particular animal. In normotensive Wistar the running wheel training did not have any effect on the LV/TL ratios. The corresponding expression of the atrial natriuretic peptide (ANP) also showed no differences (Figure 4A). The hypertension-induced myocardial hypertrophy of the LV in the non-running SHR compared to the Wistar was additionally increased by the running. This was apparent in both a significant increase in the LV/TL ratios and a significantly increased expression of ANP (Figure 4B).

Moreover, in SHR the running training induced a change in cardiac geometry which is characterized by an incipient dilatation of the LV in diastole (LVIDd) measured using echocardiography (Table 1).

**Table 1:** Functional and morphological data assessed by echocardiography.

	**Wistar sed**.		**Wistar running**		**SHR sed**.		**SHR running**	
	**3 mo**.	**6 mo**.	**3 mo**.	**6 mo**.	**3 mo**.	**6 mo**.	**3 mo**.	**6 mo**.
FS (%)	0.36 ± 0.01	0.35 ± 0.04	0.35 ± 0.01	0.39 ± 0.02^#^	0.32 ± 0.02	0.28 ± 0.01^*^	0.36 ± 0.03^*^	0.36 ± 0.05^*^
EF (Teich,%)	0.71 ± 0.01	0.70 ± 0.06	0.71 ± 0.01	0.76 ± 0.03^#^	0.65 ± 0.03	0.61 ± 0.02^*^	0.71 ± 0.04	0.72 ± 0.02^*^
MV E/A	1.55 ± 0.10	1.58 ± 0.14	1.63 ± 0.04	1.65 ± 0.15	1.63 ± 0.18	1.63 ± 0.12	1.66 ± 0.12	2.10 ± 0.14^*^^#^
IVSd (mm)	0.88 ± 0.13	0.91 ± 0.14	0.99 ± 0.20	0.90 ± 0.20	0.75 ± 0.08	0.74 ± 0.03	0.78 ± 0.07	0.88 ± 0.06^*^^#^
LVPWd(mm)	1.00 ± 0.10	0.94 ± 0.09	1.09 ± 0.08	0.94 ± 0.08^#^	0.95 ± 0.09	0.92 ± 0.07	1.05 ± 0.14	1.10 ± 0.18^*^
LVPWs(mm)	1.43 ± 0.18	1.52 ± 0.24	1.47 ± 0.14	1.64 ± 0.15	1.25 ± 0.11	1.16 ± 0.14	1.64 ± 0.25^*^	1.82 ± 0.31^*^
LVIDd (mm)	6.51 ± 0.50	6.23 ± 0.51	6.47 ± 0.24	6.14 ± 0.27	6.61 ± 0.20	6.78 ± 0.16	7.47 ± 0.33^*^	7.66 ± 0.23^*^
LVIDs (mm)	4.16 ± 0.35	4.08 ± 0.50	4.13 ± 0.09	3.79 ± 0.31	4.58 ± 0.22	4.85 ± 0.18	4.82 ± 0.33	4.98 ± 0.47

FS (%): fractional shortening, EF (Teich,%): ejection fraction, MV E/A ratio: relationship between the E and A waves, IVSd (mm): end-diastolic interventricular septum thickness, LVPWd (mm): left ventricular posterior wall thickness in diastole, LVPWs (mm): left ventricular posterior wall thickness in systole, LVIDd (mm): left ventricular internal diameter in diastole, LVIDs (mm): left ventricular internal diameter in systole. Data are means ± S.D. of n = 6 animals. Wistar/SHR sed.:

* p < 0.05 vs. 3 mo. sed., Wistar/SHR running: ^*^p < 0.05 vs. sed.,

# *p < 0.05 vs. 3 mo. running*.

### Regulation of reactive oxygen species in the left ventricular myocardium

**Figure 5:**
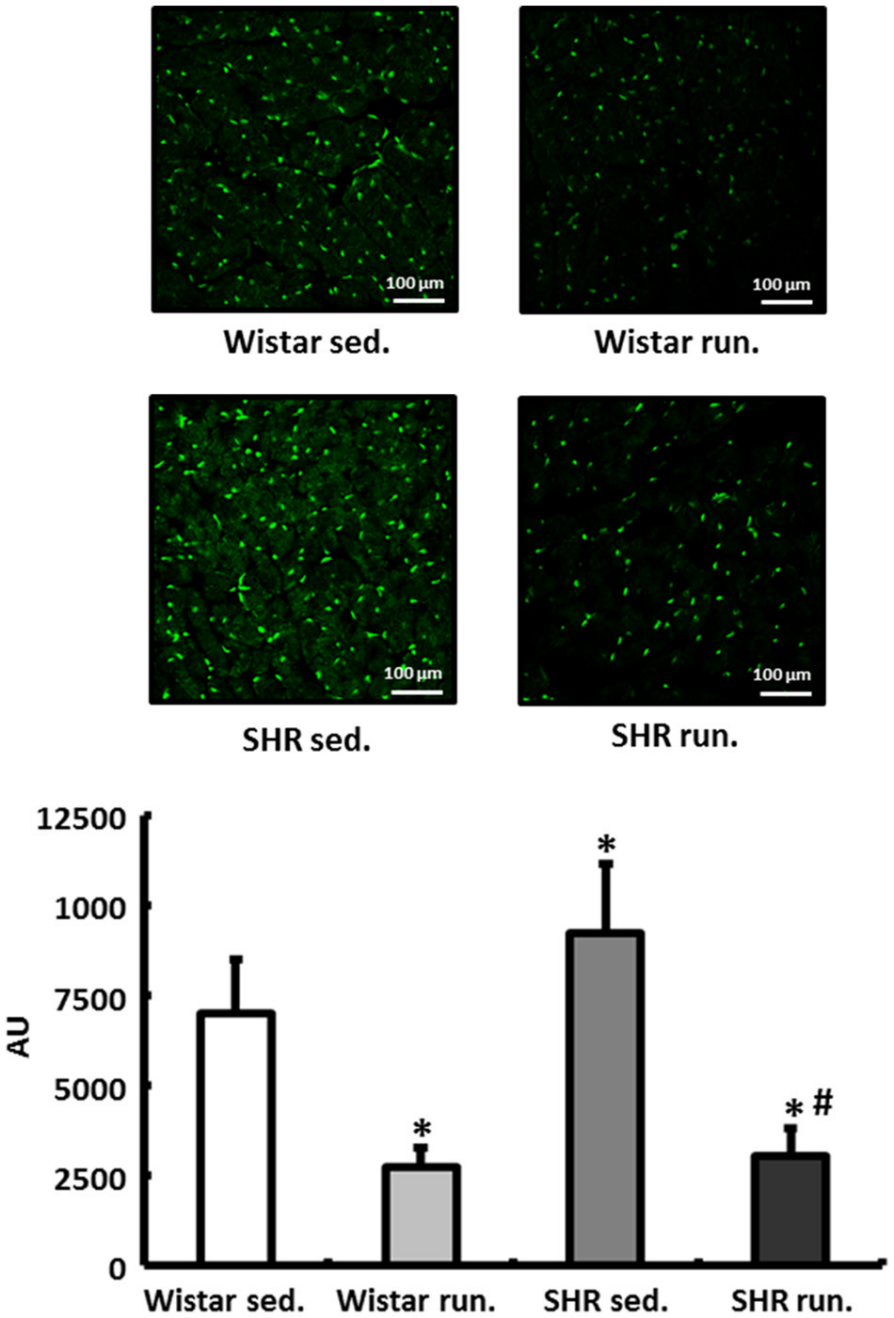
Regulation of reactive oxygen species in the LV. DHE staining was performed to measure the formation of $O_{2}^{-•}$ in heart tissue from the LV of all treatment groups. Slides were imaged by fluorescence microscopy using an excitation wavelength of 488 nm and an emission wavelength of 540 nm. Data are means ± S.D. of *n* = 4 hearts. ^*^p < 0.05 vs. Wistar sed., ^#^*p* < 0.05 vs. SHR sed.

In the fluorescence microscopy images of the dihydroethidium (DHE) staining, the hypertensive LV of non-running SHR had an elevated radical load compared to normotensive Wistar rats. The 6-months' of running training led to a significant reduction in the concentration of $O_{2}^{-•}$ in the myocardium of the LV in both the SHR and the Wistar rats (Figure 5).

### Effects of running training on the remodeling of the extracellular matrix

**Figure 6:**
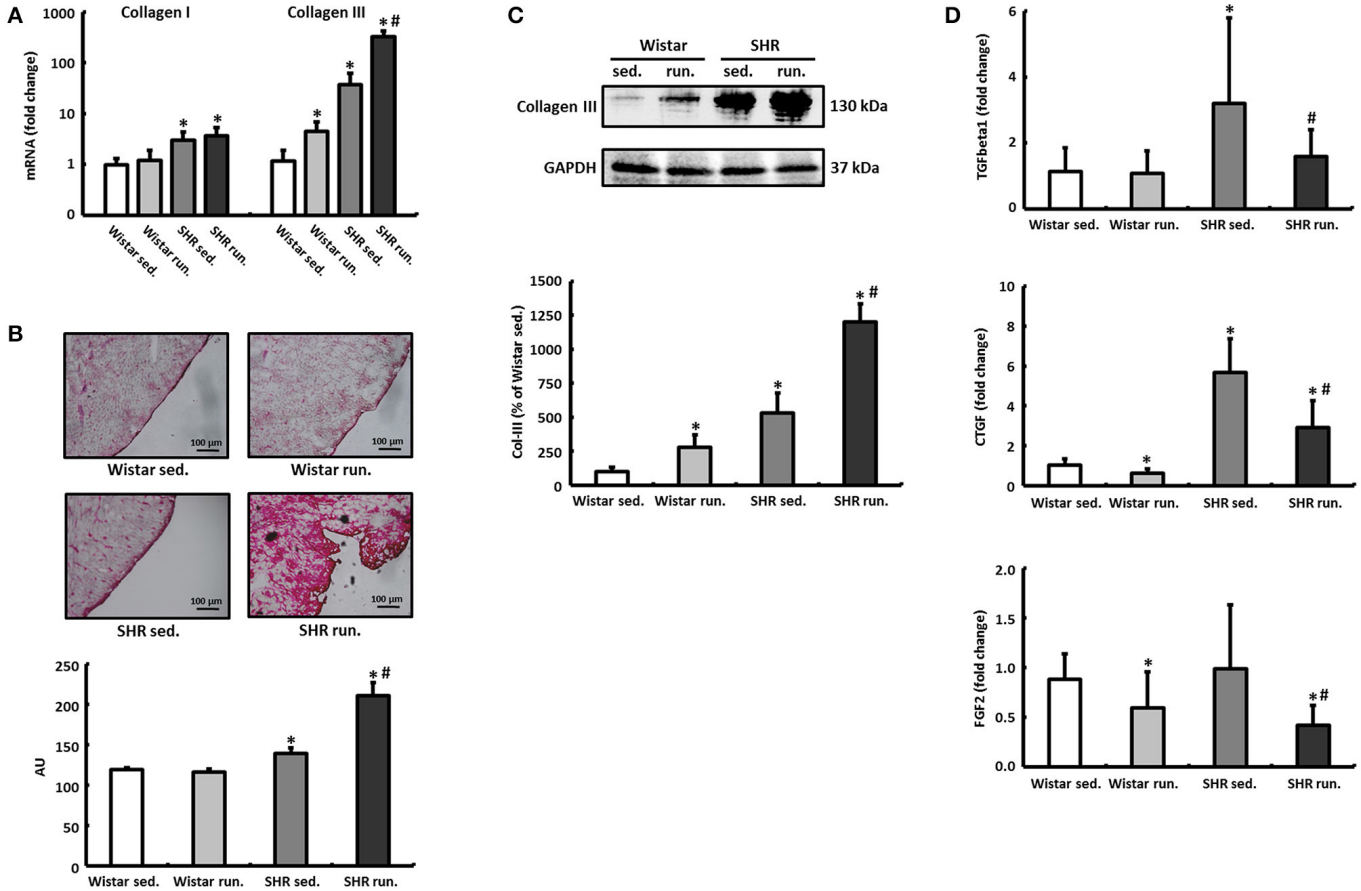
Remodeling of the extracellular collagen matrix. **(A)** The running training did not affect the expression of Col-I but markedly increased the expression of Col-III notably in SHR. **(B,C)** PCR results for collagen could be verified subsequently using both Sirius Red staining of histological sections and western blot. **(D)** The expression of pro-fibrotic factors (TGF-β1, CTGF, and FGF2) was reduced in both exercise groups. Data are means ± S.D. of *n* = 6 animals. ^*^p < 0.05 vs. Wistar sed., ^#^*p* < 0.05 vs. SHR sed.

The left ventricular expression of the extracellular matrix proteins collagen I (Col-I) and collagen III (Col-III) and the fibrosis-associated proteins TGF-β1, connective tissue growth factor (CTGF) and fibroblast growth factor-2 (FGF2) was quantified using real-time RT-PCR. The running training did not have any effect on the relative expression of Col-I in either the Wistar or the SHR. For Col-III, on the other hand, a 4.5-fold induction in the LV of trained Wistar was detected compared to their non-running controls. The SHR showed per se a 37-fold higher expression of Col-III that was increased by the running training to 326-fold (Figure 6A).

The massive upregulation of Col-III in the SHR runners could subsequently be confirmed using Sirius Red staining and western blotting (Figures 6B,C). TGF-β1, CTGF and FGF2 play a critical role in the induction of extracellular matrix proteins and thus for the development and progression of cardiac fibrosis. However, in contrast to the results for increased Col-III deposition, the running wheel training led to a significant reduction in the pro-fibrotic factors examined in runners compared to non-running controls (Figure 6D).

The observed expression pattern, and especially the selective induction of Col-III, raised the question of what mechanisms are responsible for this kind of cardiac remodeling.

### Induction of LOX and its effect on cardiac remodeling

The primary function of LOX, a copper-dependent amine oxidase, is extracellular polymerisation of monomeric matrix proteins. Intracellularly, LOX affects cell adhesion and migration as well as specifically modifying the gene expression of the Col-III isoform (Rodríguez et al., 2008).

**Figure 7:**
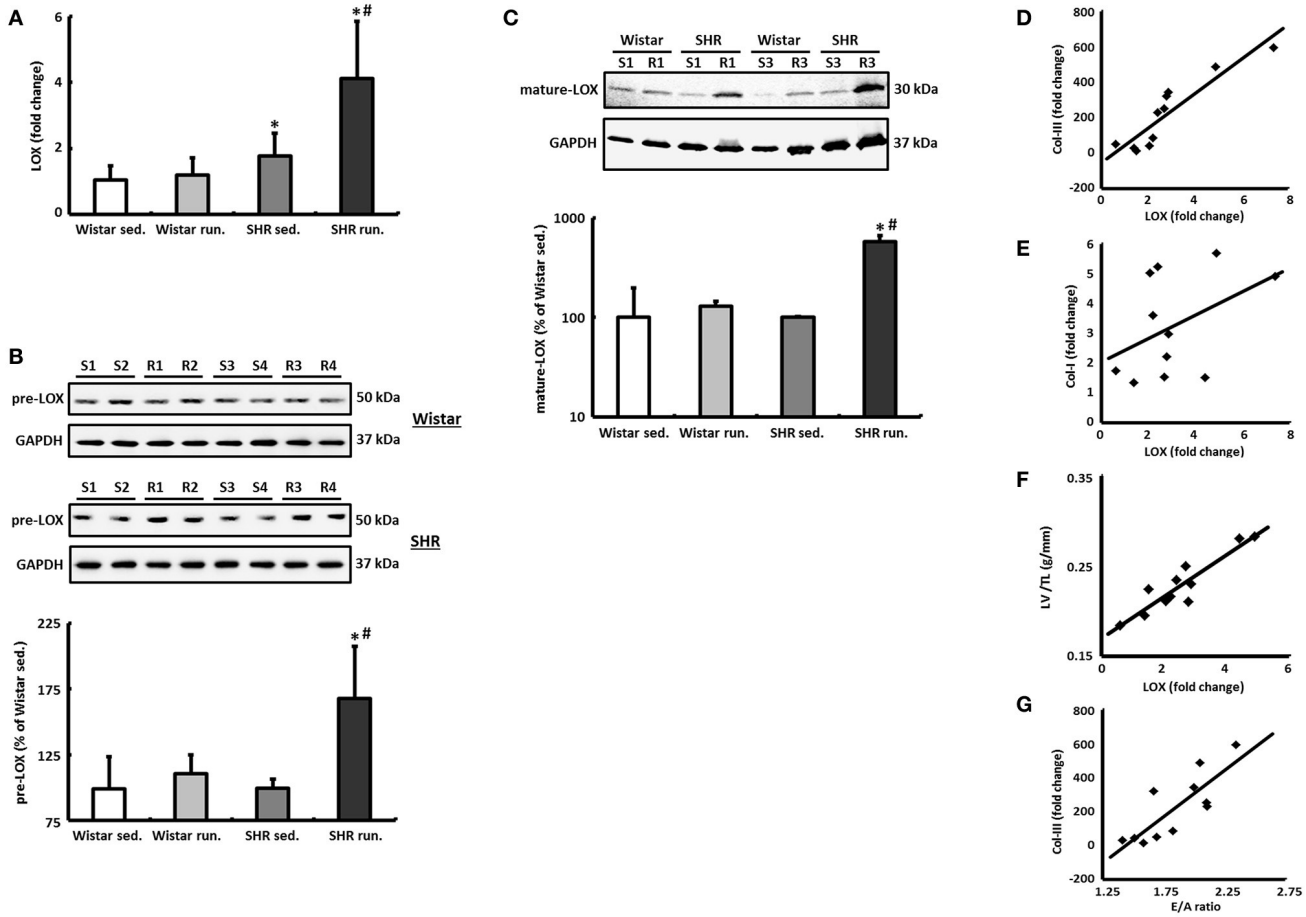
LOX as a critical risk factor for cardiac remodeling during a 6-months' aerobic running training. **(A)** The mRNA expression of LOX was analyzed in tissue from the LV using real-time RT PCR. The expression of HPRT was used for normalization. **(B,C)** Representative immunoblots and densitometric analysis of the pre-LOX (50 kDa) and the catalytically active mature LOX (30 kDa). Immunoblot bands were all normalized to GAPDH (S, sed.; R, run.). **(D–F)** A significant correlation was found between LOX expression and the expression of Col-III (*R*^2^ = 0.83) as well as the LV/TL ratio (*R*^2^ = 0.84) in SHR. However, LOX does not correlate with the mRNA expression of the Col-I isoform (*R*^2^ = 0.18). **(G)** There is also a significant correlation between the E/A ratio and the Col-III expression in SHR (*R*^2^ = 0.66). Data are means ± S.D. of *n* = 6 animals. ^*^p < 0.05 vs. Wistar sed., ^#^*p* < 0.05 vs. SHR sed.

The left ventricular mRNA expression of LOX is increased by the running training only by a moderate 12 ± 4% in the hearts of trained Wistar rats compared to their controls and does not reach the significance level. The LOX expression in the non-running SHR was already significantly above the level of the normotensive controls at 68 ± 24% and was increased by a further 132 ± 48% as a result of the running training (Figure 7A).

In parallel to the mRNA, in the SHR running group a 69 ± 22% increase in the pre-LOX detected at 50 kDa was also identified in the Western blot. For the Wistar running group again only a very moderate increase of 12 ± 2% was measured (Figure 7B).

The band of catalytically active mature LOX was detected with a molecular weight of about 30 kDa. In the Wistar runners the concentration of this fragment only increased by 29 ± 3% while in the SHR runners we observed an increase of 478 ± 72% (Figure 7C).

In both the SHR groups there is a significant correlation between LOX expression and the expression of the Col-III isoform as well as the LV/TL ratio. LOX does not correlate with the mRNA expression of Col-I, however (Figures 7D–F).

### Regulatory mechanisms for LOX in cardiac fibroblasts

During the induction and progression of cardiac fibrosis involving LOX, the glycoprotein SPP1 (also known as osteopontin or OPN) is considered to play an important role in the regulation of LOX (López et al., 2013).

**Figure 8:**
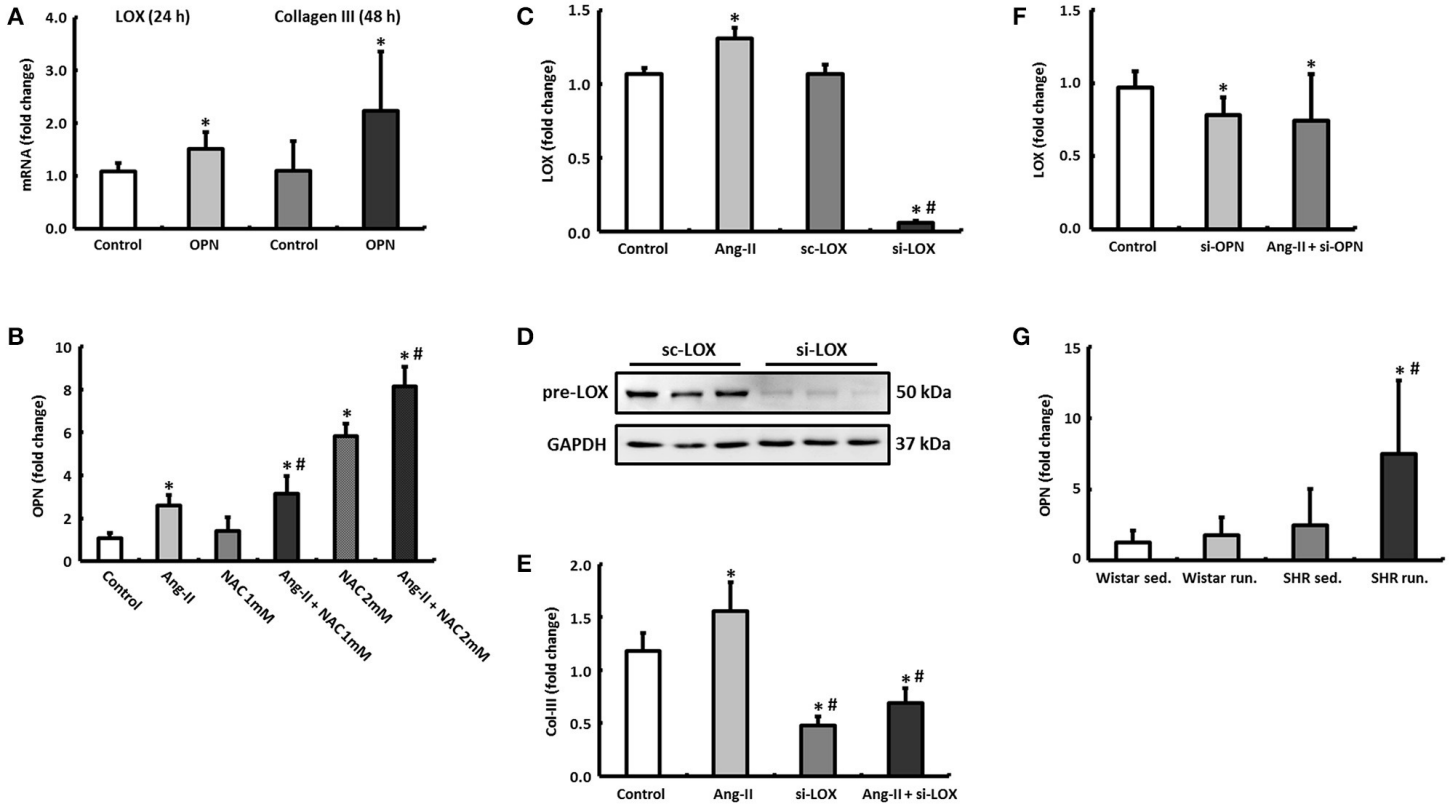
Regulatory mechanisms in cardiac fibroblasts. **(A)** By incubating cardiac fibroblasts with OPN, the mRNA expression of LOX and Col-III increased after 24 and 48 h, respectively. **(B)** OPN expression in cardiac fibroblasts could be induced by Ang-II and NAC. **(C–E)** Ang-II increased the expression of LOX and Col-III, however, Col-III expression depends on the presence of LOX (si-LOX, siRNA against LOX; sc-LOX, control siRNA). Data are means ± S.D. of *n* = 6 hearts. ^*^p < 0.05 vs. control, ^#^*p* < 0.05 vs. Ang-II. **(F)** Downregulation of OPN reduced the expression of LOX in both unstimulated and Ang-II stimulated cells after 48 h (si-OPN, siRNA against OPN). Data are means ± S.D. of *n* = 6 hearts. ^*^p < 0.05 vs. control. **(G)** OPN mRNA was primarily elevated in left ventricular tissue of trained SHR. The expression of HPRT was used for normalization. Data are means ± S.D. of *n* = 6 animals. ^*^p < 0.05 vs. Wistar sed., ^#^*p* < 0.05 vs. SHR sed.

By incubating rat cardiac fibroblasts with OPN, the expression of LOX initially increased significantly after 24 h followed by an increase in the expression of Col-III after a further 24 h (Figure 8A).

The expression of OPN could in turn be induced by stimulating the fibroblasts with angiotensin II (Ang-II). If the antioxidant effects of running training that have been described previously were simulated in fibroblast cultures by N-acetyl-L-cysteine (NAC), not only is the Ang-II effect potentiated depending on the NAC concentration but at a higher concentration (2 mM), NAC itself exerted a stimulating effect on the OPN expression (Figure 8B).

Ang-II directly induced (via OPN) the expression of LOX after 48 h (Figure 8C). By pre-incubating the fibroblasts with siRNA against LOX, the expression of LOX both at an mRNA and protein level was reduced by more than 90% (Figures 8C,D). This means that the expression of Col-III, which was already reduced at the basal level, could no longer be induced by Ang-II either (Figure 8E). However, knocking down OPN reduced the expression of LOX both at basal conditions and under Ang-II stimulation (Figure 8F).

The left ventricular express of OPN was elevated in both running groups compared to their controls (Wistar: +37.5%, n.s. or SHR +302.4%, *p* = 0.04) and thus conformed to the expression pattern for LOX (Figure 8G).

### Functional consequences of the structural remodeling process

Cardiac morphology and function were assessed using echocardiography as check-ups after 3 months and at the end of the experiment. The systolic left ventricular function was characterized using fractional shortening (FS) determined along the long parasternal axis in M mode. For the Wistar there was a significant improvement in the FS only in the second part (3rd to 6th month) of the training period while there was an improvement in the FS in the SHR after just 3 months (Table 1). As a surrogate of diastolic function the ratio of the early diastolic filling and the late atrial filling (E/A ratio) was used. For the Wistar no change was observed in either the running or the non-running animals over the entire training period. While the non-running SHR also maintained a ratio at the level of the Wistar, in the trained SHR the E/A ratio—along with a significant reduction in A max velocity—increased above the value of 2 in the second half of the training period, resembling a pseudonormalization and thus may indicate a functionally relevant restrictive filling disorder (Table 1). The significant correlation between the E/A ratio and the Col-III expression indicates a causal involvement of the Col-III isoform in the diagnosed relaxation disorder in the trained SHR (Figure 7G).

## Discussion

Following 6-months' voluntary training on a running wheel young Wistar and pre-hypertensive SHR were analyzed for various markers of cardiac remodeling. A rise in the mitochondrial respiration was observed in the animals in both running groups in the cardiac muscle and the skeletal musculature. The running training also reduced not only the left ventricular concentration of $O_{2}^{-•}$ in the Wistar rats and SHR but also the expression of genes that are involved in the induction and progression of heart failure. The training led to a continuous reduction in the HR in both running groups and improved the systolic function of Wistar and SHR.

On the downside, however, the running training induced a myocardial hypertrophy in the SHR runners as well as a marked Col-III specific fibrosis that was reflected in a functionally relevant filling disorder in the echocardiograms. A massive upregulation of OPN and LOX has been identified as the underlying cause of the subsequent remodeling of the left ventricular myocardium in hypertensive runners. The findings also show that a 6-months' voluntary endurance training does not have any effect on the changes over time or on the absolute systolic or diastolic BP throughout the entire observation period in Wistar rats or SHR.

The key experimental finding of this study that is described here for the first time is the identification of LOX as a critical risk factor for cardiac remodeling during a 6-months' aerobic endurance training.

Collagen isoforms are first secreted as monomers into the extracellular space and only form stable polymers as a result of post-translational modification. The initial step to form these aggregates, oxidative deamination of epsilon amino groups, is carried out by the catalytically active form of LOX (Rodríguez et al., 2008). An elevated expression of cardiac LOX subsequently results in an increased network of collagen (and elastin) isoforms and has already been described as the reason for the increase in ventricular stiffness with the resultant symptoms of heart failure (López et al., 2010). Intracellularly, pre-LOX also affects the gene expression of the Col-III isoform by specifically activating the COL3A1 promoter (Giampuzzi et al., 2000). Cardiac fibrosis in the SHR was not yet functionally relevant after 3 months of the experimental setup but after 6 months the E/A ratio increased to a value ≥2, corresponding to stage 3 of a restrictive filling disorder (Oh et al., 2011). Both properties of LOX, the increased network of matrix proteins and the elevated Col-III expression, must be considered responsible for the diastolic functional disorder. Even the moderate increase in the LOX expression in the Wistar runners increased Col-III production by 4.5 times but did not have any functional consequences.

OPN, an acidic, highly phosphorylated glycoprotein for which clinically relevant pleiotropic effects within the cardiovascular system have previously been described, is considered a direct stimulus for increasing LOX expression (Collins et al., 2004; López et al., 2013). Combined with the *in vitro* experiments carried out here, the situation in the heart of runners can be described as follows: adaptive, predominantly antioxidative, mechanisms prevent “classic” remodeling based on oxidative stress and TGF-β in the myocardium of the runners. Instead, activation of the sympathetic nervous system or RAAS that is caused by running and hypertension induces via Ang-II the expression of OPN and subsequently of LOX which specifically activates the Col-III promoter and increases the rigidity of the ventricle due to cross-linking (Matsui et al., 2004; Nakayama et al., 2011; Lorenzen et al., 2015).

The pathomechanism described above was also reflected in the training performance of the animals: While normotensive running animals completed a consistently steady training workload, the weekly distance ran by the SHR declined continuously and at the end of the experiment the distance was about 45% below the baseline values in the first month of training.

In contrast to the described antihypertrophic effects or delayed re-expression of fetal genes, the SHR in this study developed a cardiac hypertrophy during their 6-months' training period, which was accompanied by an accelerated upregulation of the ANP. This development corresponds to the cardiac changes previously observed to an even greater degree in older hypertensive animals (Schultz et al., 2007; da Costa Rebelo et al., 2012). However, it has already been demonstrated for OPN that it is causally involved in the development of cardiac hypertrophy, meaning that the development of left ventricular hypertrophy in the SHR runners must also be taken into account as a consequence of the above-described “non-classic” remodeling (Graf et al., 1997; Xie et al., 2004). The normotensive Wistar did not differ from their non-running controls in terms of heart weight or ANP expression, however. The systolic pump function measured using echocardiography also improved during the training period in the animals in both running groups. These results indicate that the additional running-induced hypertrophy in the SHR up to this point can be attributed to the stage of adaptation. Moreover, incipient left ventricular dilatation, a well-recognized precursor of congestive heart failure, could be almost completely compensated in view of the improved EF. To what extent physical training contributes to an improved EF or prevents the development of systolic heart failure is still largely unknown. Important factors of influence are the animal model used, the age of the experimental animals, and the duration and intensity of the training. In this context, beneficial effects are predominantly described in young experimental animals combined with a “moderate” training workload (Garciarena et al., 2009). On the other hand, Schultz et al. (2007) reported a deterioration of systolic function in older hypertensive animals after a training program of high physical intensity.

Arterial hypertension is a primary risk factor that is directly associated with the pathogenesis of coronary heart disease, myocardial infarction, and heart failure. Systematic antihypertensive therapy enables a critical reduction in the morbidity and mortality of patients. In addition to a drug intervention, a fundamental component of systematic antihypertensive therapy in general is the recommendation for patients to appropriately adjust their lifestyle: This includes adopting a low-fat and low-sodium diet, stopping smoking, and incorporating more physical activity (Hagberg et al., 2000).

However, the extent to which physical training alone contributes to lowering BP can only be estimated with difficulty because a number of factors change along with the change in lifestyle. In an animal model it is possible to specifically control these factors. The current study therefore takes the approach of defining physical training alone as the test condition.

Compared to “moderate” swimming or treadmill training, the weekly running performances achieved by the Wistar rats and the SHR in this study are indicative of a high training performance or intensity. However, voluntary training of rats is performed as an intermittend, noctural running pattern which consists of 100–150 individual running bouts per 24 h. Typically, each revolution/bout takes a maximum of 3 min (Overton et al., 1986; Rodnick et al., 1989). This means that the average running distance of ~6 km per day is distributed over many hours in intervals with longer recovery phases. Thus, voluntary wheel-running in rodents is thought to be an appropriate model of aerobic exercise in humans (Yasumoto et al., 2015). In light of these factors, voluntary training with a running wheel allows the rat a largely natural running behavior, and thus represents the most physiological model. However, the significant increase in BW in running SHR can be mainly attributed to higher food consumption during exercise and have already been identified as a characteristic specific to SHR (Gordon et al., 2016).

The running wheel as a training device enables the individual animal to complete an individual training workload according to its willingness to run. The naturally higher physical activity of the SHR compared to the Wistar is thus reflected in a significantly higher weekly running distance and time (Sagvolden et al., 1993). Nevertheless, the individual desired functional and metabolic adaptation reactions of the training on SHR and Wistar are highly comparable despite the varying running performances as described in the following sections.

At the end of the experimental period, the function of the complexes I and II were examined using microrespiratory tests on permeabilized muscle fibers from the LV and the soleus muscle. In the LV the activity of the pyruvate-dependent complex I respiration increased in the animals in both running groups while no change was detected in the succinate respiration of complex II. The resulting large increase in the succinate-related pyruvate respiration (SRPR) together with unchanged expression of PGC-1 α and NRF1 (mRNA, measured by real time PCR), which are involved directly in the biogenesis of mitochondria as transcriptional cofactors, is indicative mainly of an increase in the activity of complex I and less of an increase in the number of mitochondria (Ventura-Clapier et al., 2008).

A clear increase in the complex I activity was also verified in the skeletal musculature for both running groups. Unlike the LV, the succinate respiration also increased in the soleus muscle in Wistar and SHR. The resulting increase in the SRPR resulting from these values corresponds to the qualitative changes in individual respiratory chain complexes in the skeletal musculature described by Daussin et al. as a result of a two-year defined training program of moderate to high training intensities (Daussin et al., 2008).

The lowering of the resting HR in both running groups also contributes to an improvement of the cardiac energy metabolism due to a reduction in both ventricular work and myocardial oxygen consumption.

The causal involvement of the cytokine TGF-β1 in the induction and progression of heart failure has already been well documented. It is also critically involved in the development and maintenance of cardiac fibrosis (Rosenkranz, 2004). The training program used in this study did not have any effect on the left ventricular expression of TGF-β1 in the normotensive Wistar but did lead to a significant reduction in the SHR. The expression of Col-III was induced in both running groups while that of Col-I was not affected by the running training. Thus, the Col-III specific fibrosis developed independently of TGF-β1 as well as CTGF or FGF2 which were also expressed less in both running groups. CTGF, however, is causally involved in pathological fibrosis and also contributes to heart failure development (Koshman et al., 2013; Szabó et al., 2014). FGF2 mainly promotes cardiac hypertrophy and cardiac fibrosis (Nusayr et al., 2013). The downregulation of these three genes, the expression of which is fundamentally affected by radical metabolism, can also be explained by the antioxidant effects of the running training that are revealed by a reduction in the production of $O_{2}^{-•}$ in the left ventricular tissue (Dekleva et al., 2017).

## Conclusion

Adaptive responses in both normotensive Wistar rats and pre-hypertensive SHR that improved the metabolic and functional remodeling of the myocardium were induced by 6 months' running training but with no effect on either the temporal development or the absolute value of the BP.

**Figure 9:**
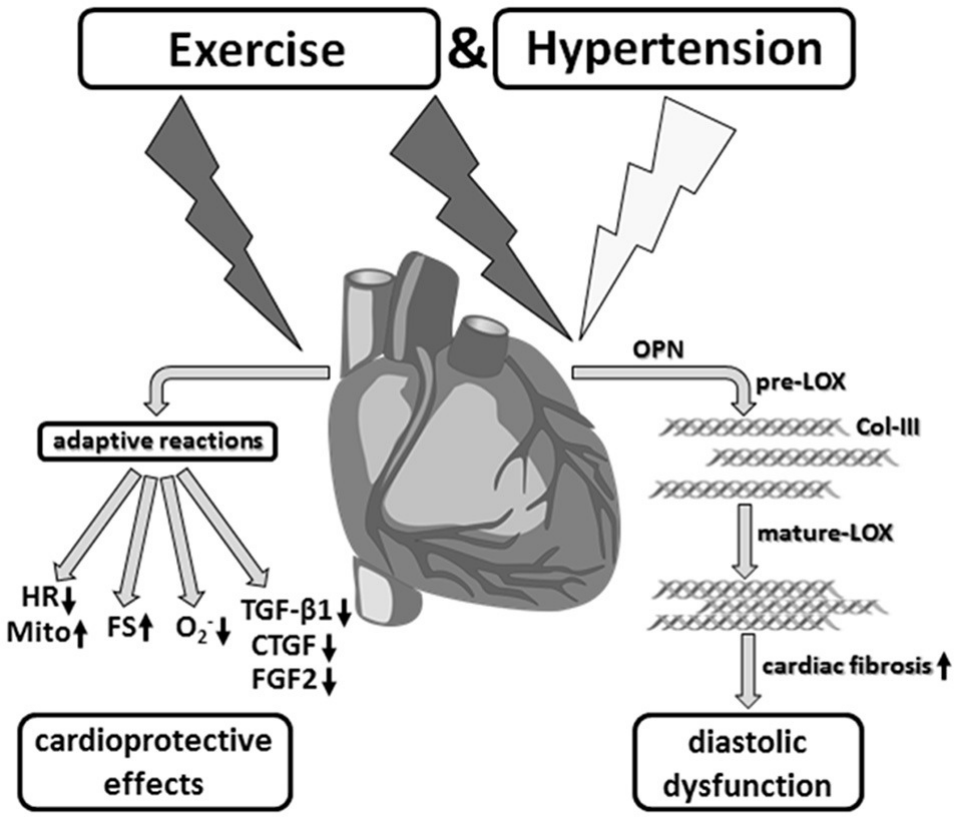
Aerobic endurance training in the concomitant presence of hypertension. Despite desired functional and metabolic adaptation reactions, running training in combination with arterial hypertension induced a myocardial hypertrophy as well as significant fibrosis, and thus, a diastolic functional disorder, which critically affects the prognosis in the medium to long term.

At the same time, however, during sustained running stress these cardioprotective adaptive mechanisms contribute to the development of non-classic remodeling of the left ventricular myocardium that is characterized by induction of LOX, Col-III specific fibrosis and diastolic dysfunction (Figure 9).

## Prospects

Analogous to the indication, dosage and duration of pharmacotherapy administered in accordance with the guidelines, developing and monitoring an individual training program are essential to reduce cardiovascular risk factors for existing hypertension. The results of this study show that aerobic endurance training with existing high BP can be of therapeutic benefit but is also associated with corresponding risks. Consistent with the conclusions drawn by Allesøe et al., further studies are essential to establish sports and physical activity as a safe preventive and therapeutic option for cardiovascular diseases (Allesøe et al., 2016; Williamson et al., 2016).

## Study limitations

This study investigates the impact of voluntary wheel-running as a sole intervention on the cardiac remodeling in terms of functional, structural, and metabolic adaptation reactions using female Wistar rats and SHR. Both male and female rats have been used frequently in studies examining the effects of exercise on the cardiovascular system. However, the estrous cycle and the ovarian hormones are likely to be responsible for well-reported differences in voluntary running performance in female compared to male rats. Thus, it could be shown that running wheel activity increased significantly during the night of proestrus. Although the influence of stage of estrous cycle are likely to be averaged out during 6 months' experimental period within the four treatment groups, we cannot rule out that male Wistar rats and SHR differ by the type of cardiac remodeling in response to 6-months' voluntary training on a running wheel.

Furthermore, the strong correlation between LOX expression and the expression of the Col-III isoform as well as Col-III expression and the E/A ratio indicates a causal involvement of LOX in the diagnosed relaxation disorder in the trained SHR. However, the extent of collagen cross-linking has not been determined directly in cardiac tissue.

## Author contributions

RS and KS: Conceptualization, Supervision, Methodology, Formal analysis, Writing–Original Draft, Funding acquisition; AH, Rd, SS, and LL: Methodology, Investigation; BN and SR: Methodology, Investigation, Formal analysis.

### Conflict of interest statement

The authors declare that the research was conducted in the absence of any commercial or financial relationships that could be construed as a potential conflict of interest.

## Abbreviations

Ang-II: Angiotensin II
ANP: atrial natriuretic peptide
BP: blood pressure
BW: body weight
Col-I: collagen I
Col-III: collagen III
CTGF: connective tissue growth factor
DHE: dihydroethidium
EF: ejection fraction
FGF2: fibroblast growth factor-2
FS: fractional shortening
HR: heart rate
IHD: ischaemic heart disease
LOX: lysyl oxidase
LV: left ventricle
NAC: N-acetyl-L-cystein
Nrf-1: nuclear respiratory factor-1
OPN: osteopontin
$O_{2}^{-•}$: superoxide
PGC-1α: peroxisome proliferator activated receptor gamma coactivator-1α
RAAS: renin-angiotensin system
SHR: spontaneously hypertensive rat
TGF-β1: transforming growth factor-β1
TL: tibia length.
