# Supplementary material for: Effects of 6-months' Exercise on Cardiac Function, Structure and Metabolism in Female Hypertensive Rats–The Decisive Role of Lysyl Oxidase and Collagen III
Source: Front Physiol. 2017 Aug 3;8:556. doi: 10.3389/fphys.2017.00556 (PMC5541302; doi:10.3389/fphys.2017.00556)
Supplement: Supplementary file 1 [file DataSheet1.DOCX]

**Supplementary Material**


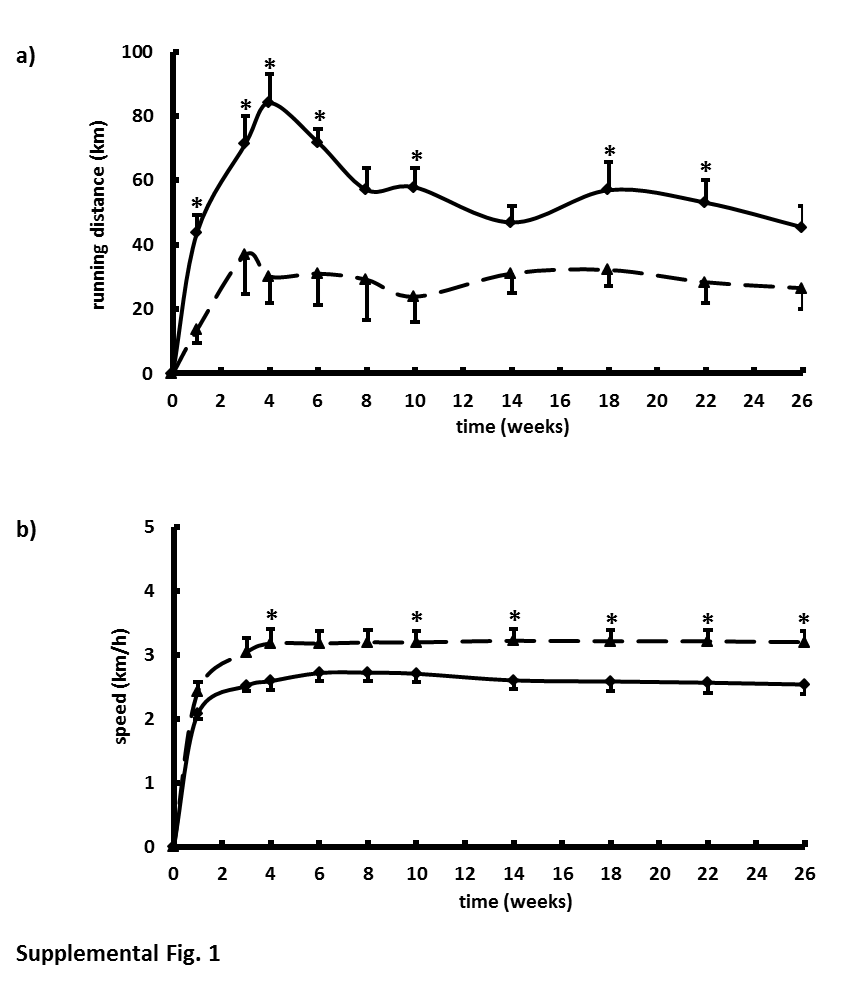


**Supplemental Fig. 1: Training data of SHR and Wistar over the entire experimental period.** a) Average weekly running distance for pre-hypertensive SHR (represented by a solid line) and Wistar (represented by a broken line). b) Lines represent an average running speed expressed in km per hour (km/h) for both exercise groups. Data are means±S.D. of n=6 animals. *, p< 0.05 vs. Wistar.

|  | GenBank accession | annealing  temperature (°C) | Forward Primer | Reverse Primer |
| --- | --- | --- | --- | --- |
| HPRT | NM_012583 | 63.0 | CCA GCG TCG TGA TTA GTG AT | CAA GTC TTT CAG TCC TGT CC |
| ANP | NM_012612 | 58.0 | ATG GGC TCC TTC TCC ATC AC | TCT TCG GTA CCG GAA GCT G |
| TGF-β1 | NM_021578 | 61.0 | ATT CCT GGC GTT ACC TTG G | CCT GTA TTC CGT CTC CTT GG |
| CTGF | NM_022266 | 60.0 | TGT GAA GAC CTA CCG GGC TA | TTC ATG ATC TCG CCA TCG GG |
| FGF2 | NM_019305 | 60.0 | TCC ATC AAG GGA GTG TGT GC | TCC GTG ACC GGT AAG TGT TG |
| Col-I | NM_053304 | 62.0 | GCG AAC AAG GTG ACA GAG | CCA GGA GAA CCA GCA GAG |
| Col-III | NM_032085 | 55.0 | TGG AGT CGG AGG AAT G | GCC AGA TGG ACC AAT AG |
| LOX | NM_017061 | 60.0 | ACA ACC GCA CTG CCT CTG CC | GCC TTG AGG CTC CAT CGC CG |
| OPN | NM_012881 | 60.0 | GAT GAC GAC GAC GAT GAC GA | GCT GGC AGT GAA GGA CTC AT |

**Supplemental Tab. 1:** GenBank accession numbers, annealing temperatures and sequences of PCR primers used in this study.
